# Cost-Effectiveness of Nivolumab Immunotherapy vs. Paclitaxel or Docetaxel Chemotherapy as Second-Line Therapy in Advanced Esophageal Squamous Cell Carcinoma in China

**DOI:** 10.3389/fpubh.2022.923619

**Published:** 2022-06-29

**Authors:** Ying-tao Lin, Tian-xiu Liu, Jian Chen, Chang Wang, Ying Chen

**Affiliations:** ^1^Department of Drug Clinical Trial Administration Office, Fujian Medical University Cancer Hospital, Fujian Cancer Hospital, Fuzhou, China; ^2^Department of Thoracic Radiotherapy, Fujian Medical University Cancer Hospital, Fujian Cancer Hospital, Fuzhou, China; ^3^Department of Surgical Oncology, Fujian Medical University Cancer Hospital, Fujian Cancer Hospital, Fuzhou, China; ^4^Department of Medical Oncology, Fujian Medical University Cancer Hospital, Fujian Cancer Hospital, Fuzhou, China

**Keywords:** cost-effectiveness, partitioned survival model, therapy, drug acquisition cost, esophageal squamous cell carcinoma

## Abstract

This study aimed to evaluate and compare nivolumab's cost-effectiveness with chemotherapy in patients with advanced esophageal squamous cell carcinoma from the Chinese healthcare system perspective. To this end, the researchers utilized a partitioned survival model with three mutually exclusive health stages. The characteristics of the patients used as inclusion and exclusion criteria in this model were the same as those used for patients with advanced esophageal squamous cell carcinoma in the ATTRACTION-3 study. The ATTRACTION-3 trial, which took place between January 7, 2016 and November 12, 2018, also yielded important clinical data. Data on medical and economic preferences were collected from real-world clinical practices. Costs, quality-adjusted life years, and incremental cost-effectiveness ratio were calculated for the two therapy options. The model uncertainty was investigated using a deterministic and probabilistic sensitivity analysis. When compared to chemotherapy, nivolumab was linked with an increase of 0.28 quality-adjusted life years with an increased cost of US$ 36,956.81 per patient in the base case analysis of a hypothetical sample of 419 patients. The incremental cost-effectiveness ratio in the deterministic sensitivity analysis was US$ 132,029.46/quality-adjusted life year, with a 48.02% probability of being cost-effective at willingness-to-pay thresholds of US$ 132,029.22/quality-adjusted life year. The incremental cost-effectiveness ratio remained greater than US$ 80,000/quality-adjusted life year in the deterministic sensitivity analyses. To be more cost-effective and remain below the threshold of 37,653 US$/quality-adjusted life year, which the Chinese population can afford, nivolumab's price would have to be lowered sharply by 53.50%. Nivolumab is clinically beneficial but not cost-effective when compared to chemotherapy. A substantial reduction in nivolumab's drug acquisition cost would be necessary to make it cost-effective for immunotherapy.

## Introduction

Esophageal cancer is one of the seven major malignant tumors worldwide and is the sixth leading cause of mortality among all malignancies (1, 2). Esophageal cancer incidence, prevalence, and histological type vary among geographic regions. For instance, North America and Western Europe have the highest rates of esophageal cancer, (3, 4) where its most common subtype is adenocarcinoma. Meanwhile, in Asia, including China, Japan, and Korea, esophageal squamous cell carcinoma (ESCC) is more common (5, 6). Advanced esophageal cancer is a rapidly fatal disease (7). Approximately 40% of patients with esophageal cancer are diagnosed when the disease is advanced, and the median survival time is 8–10 months. The 5-year survival rate is predicted to be below 5%. Furthermore, patients with advanced esophageal cancer have limited options for second-line treatments, (8, 9) with no accepted standard of care, although paclitaxel, docetaxel, or irinotecan are used (10–12). Publications summarizing data from retrospective analyses have reported that the median survival and overall response rate are comparable among paclitaxel, docetaxel, and irinotecan (13–15). In addition, Nivolumab, an anti-programmed death 1 (PD-1) inhibitor, has shown antitumor activity in patients with advanced esophageal cancer (16, 17). ATTRACTION-3, (18) a published clinical trial of nivolumab, reported clinical efficacy of treatment in terms of longer overall survival (OS) compared with chemotherapy using paclitaxel or docetaxel.

Recently, given their antitumor activity, PD-1 inhibitors are being used in the treatment of several types of squamous cell tumors (19–21). This treatment comes at a high cost and increases patients' financial burden (22). Though a therapy's clinical effectiveness is desirable, its economic cost is an important consideration for healthcare policymakers while selecting treatment options. If the cost of PD-1 inhibitors is high, it may outweigh the benefit of their antitumor effect. Based on the ATTRACTION-3 trial data, our study attempted to assess the cost-effectiveness of nivolumab immunotherapy and paclitaxel/docetaxel chemotherapy treatment alternatives by measuring and comparing therapy costs and effectiveness from the perspective of the Chinese society.

## Materials and Methods

### Target Population

This study was conducted at Fujian Medical University Cancer Hospital, Fuzhou, China. The study was designed by referring to the International Council for Harmonization E6 guidelines for Good Clinical Practice, the Declaration of Helsinki principles, and applicable laws and regulations. The reporting criteria of the Consolidated Health Economic Evaluation Reporting Standards were followed when writing the economic evaluation section (23).

The target population in the model was the same as that used in the ATTRACTION-3 clinical trial. The ATTRACTION-3 trial is a global, multicenter, randomized, open-label, phase 3 study. The trial covered 90 cancer centers and hospitals across Asia, North America, and Western Europe. A total of 419 patients were recruited for this study, who received at least one cycle of the assigned therapy. From the 419 patients, 210 were assigned to receive nivolumab and 209 to receive chemotherapy (144 and 65 patients were assigned to receive paclitaxel and docetaxel, respectively). Patients included in the study were at least 20 years old and diagnosed with unresectable esophageal cancer, either squamous or adenosquamous cell carcinoma. The diagnoses were confirmed by histological or cytological features. At least one measurable lesion should have been present (a major resected lesion in the cervical or thoracic esophagus or at the esophagogastric junction). They should have had tumor progression or recurrence after the first-line treatment (including chemoradiotherapy). Other inclusion criteria were: a 0–1 Eastern Cooperative Oncology Group performance status and adequate organ function. The treatment continued until any of the following events occurred: disease progression as defined by the Response Evaluation Criteria in Solid Tumors version 1.1, the occurrence of unacceptable toxicity levels, patient withdrawal, or at the investigator's discretion.

### Model Construction

**Figure 1:**
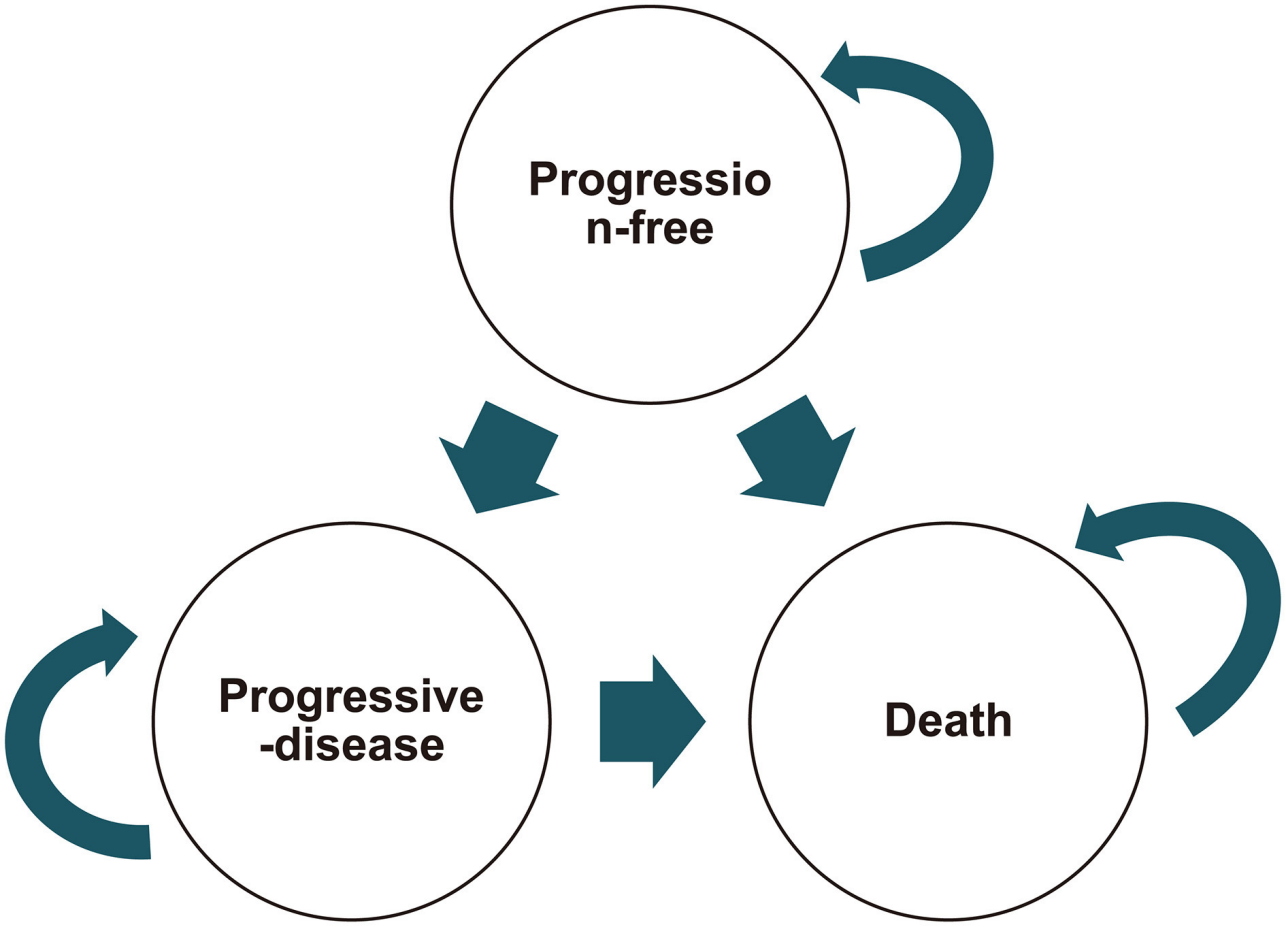
Transition dagram for partitioned survival model health outcomes.

The cost-effectiveness of treatment with nivolumab and chemotherapy with paclitaxel or docetaxel was assessed using a partitioned survival model (24) based on the ATTRACTION-3 trial data. This model has often been used in testing medical costs and efficacy outcomes of metastatic oncology modeling (25–28). The model has three mutually exclusive health stages (Figure 1): progression-free stage (patient entered until disease progression occurred), progressive disease (PD) stage (patient was alive after the disease progression began), and terminal stage. The length of each model cycle was defined as 60 days, and the time horizon was assessed at 36 months in our model, which matched the actual progress of the ATTRACTION-3 trial. The model's key output variables were cost, quality-adjusted life years (QALYs), and incremental cost-effectiveness ratio (ICER).

### Cost

In our model, clinical costs were considered, including drug acquisition, laboratory tests, radiologic images, drug administration, disease progression visits, treatment-related adverse events (AE), and terminal costs. These costs were direct costs, which were converted to US$ at the rates prevailing in November 2021. The data on costs were collected from the National Health Commission of China, Fujian Provincial Health Commission, National Comprehensive Cancer Network Clinical Practice Guidelines in Oncology, and expert consensus.

The administered doses of nivolumab and chemotherapy were included in the drug acquisition cost. The evaluated drugs in the model included nivolumab (Bristol-Myers Squibb), paclitaxel (Bristol-Myers Squibb), and docetaxel (Aventis Pharma S. A.). The listed drug prices, obtained from the National Health Commission of the People's Republic of China in 2021, were nivolumab at US$ 718 per 4 ml: 40 mg and US$ 1, 448 per 10 ml: 100 mg; paclitaxel at US$ 77 per 5 ml: 30 mg; and docetaxel at US$ 142 per 0.5 ml: 20 mg. The dosing frequency and intensity were based on the ATTRACTION-3 trial's published data. Nivolumab's dose, administered intravenously, was 240 milligrams on day 1 of each 2-week treatment cycle (each treatment cycle lasted 6 weeks). Chemotherapy was administered with a dose of 100 mg/m^2^ of paclitaxel on day 1 of each 1-week cycle (6 weeks per cycle followed by 1 week off) or with a dose of 75 mg/m^2^ of docetaxel on day 1 of each 3-week cycle (each treatment cycle was 3 weeks). Since body surface area was not reported in the ATTRACTION-3 trial, we assumed a body surface area of 1.71 m^2^ to calculate the doses of paclitaxel and docetaxel. This body surface area was based on a mean height of 1.64 m and a mean bodyweight of 64 kg, which were the mean values of the Chinese population in 2020, as published by the National Bureau of Statistics of the People's Republic of China. Therefore, the dose of nivolumab was set at 240 mg. The mean doses of paclitaxel and docetaxel per patient in the chemotherapy group were 115 and 275 mg, respectively. The cost was determined at the patient level for all vials.

The standard charges of the Fujian Provincial Health Commission in 2021 were used to compute the expenses of laboratory testing, radiologic imaging, medicine administration, disease progression visits, and AE-related costs. Terminal costs were estimated according to the relevant legal interpretations of the Supreme People's Court in trials of personal injury compensation cases (29).

Laboratory tests and radiologic imaging costs assumed that the schedule of assessments in typical clinical trials was followed while performing these tests. Therefore, all laboratory tests and radiologic images in our model were not assumed to have been performed at the onset of treatment (first day of each model cycle). The costs of these laboratory tests and radiologic imaging were accounted for whenever they were performed as required by the treatment duration, histology, and time horizon. From 28 days before the baseline until the completion of treatment, the 12-lead electrocardiogram, Hepatitis B virus and Hepatitis C virus serology, hematology, serum chemistry, coagulation tests, urinalysis, thyroid function, tumor assessment, and pulmonary function test were conducted. Hematology, serum chemistry, 12-lead electrocardiogram, coagulation, and urinalysis were performed within 14 days before the baseline. These tests were repeated and reviewed before nivolumab, paclitaxel, or docetaxel administration. Hepatitis B virus and Hepatitis C virus serology tests, including Hepatitis B surface antigen, Hepatitis B core antibody, and Hepatitis C virus antibodies, were performed within 14 days of the baseline. Patients who were Hepatitis B surface antigen-positive were not enrolled until further definite testing with Hepatitis B virus DNA titers showed a satisfactory protective level of anti-HBs. Pulmonary function tests, including spirometry and assessment of diffusion capacity, were performed within 28 days of the baseline to determine enrollment suitability. Thyroid function tests were performed within 7 days of the baseline to determine the levels of free triiodothyronine, free thyroxine, and thyroid stimulating hormone, and were repeated three times and each time the drug was administered intravenously thereafter (nivolumab, 6 weeks; paclitaxel, 3 weeks; docetaxel, 9 weeks). Tumor assessments were performed using contrast-enhanced computed tomography scans of the neck, chest, and abdomen within 28 days of the baseline, every 6 weeks for 1 year, and every 12 weeks thereafter, until disease progression or death, whichever occurred first. For patients who could not be subjected to computed tomography because of contrast dye allergies, magnetic resonance imaging was used. For each patient, the same radiographic procedure was used throughout the study.

For nivolumab and chemotherapy, drug administration expenses were examined separately, including preventative medicine, hospitalization, nursing, and drug infusion expenditures. Patients in both arms of the trial were assumed to be routinely monitored until death, and medical examination and visit expenditures were expected to be incurred when disease progression occurred. Terminal costs were allocated when a patient died; the costs for these services were assumed to be equal in both arms. The one-time cost of a funeral by burial was characterized as the terminal cost. Our model included the ≥3-grade treatment related to AE, as reported in the ATTRACTION-3 trial. The related treatment cost calculations for the nivolumab group were derived from the National Comprehensive Cancer Network Clinical Practice Guidelines in Oncology: Management of Immunotherapy-Related Toxicities Version 4.2021 (30). The treatment cost for the chemotherapy group was based on the expert consensus of clinical practitioners.

### Utility Scores

The ATTRACTION-3 trial did not report the utility scores. Various scholars have used the reported quality-of-life data as utility scores for cost-effectiveness analyses regarding esophageal cancer treatment (31–37). There may be considerable uncertainty regarding ESCC's impact on QALYs, especially given the current uncertainty in published reports regarding the value for utility score assessment. The only realistic assumption supported by these published reports and current practices is that the utility scores in second-line esophageal cancer treatment would eventually decline as the disease progressed to death (38–40). This is because decreased functioning or worsening symptoms during and after second-line treatment is inevitable (41). Therefore, in our model, we assumed the utility score to decline linearly from progression-free survival (PFS) to the point of PD and then to the point of death. The utility score was determined to be 0.74 in PFS and 0.58 in PD (34, 37). Mortality's utility score was 0.

### Sensitivity Analyses

A deterministic sensitivity analysis (31, 42) was conducted by adjusting all the model's input parameters. Table 1 presents characteristics of the model's costs and outcome parameters. Table 2 presents laboratory tests, scans, and ≥3 grade treatment-emergent AE costs and treatment details. The discount rate for both costs and health outcomes was 5% per year, range from 0 to 8% (46). Cost of HBV and HCV serology, HBV DNA, radiologic images, and ≥grade 3 AE-related costs were based on the clinical practices estimation for value range, other parameters were changed by 20% in both directions. When one of the input parameters was altered, the others remained unchanged. A probabilistic sensitivity analysis was executed using a Monte Carlo simulation (34, 47). A total of 10,000 simulated iterations were run. Each time, a random sample was taken from the distributions of all the parameters. The parameter categories were used to make assumptions about distributions, the cost parameters were assumed to Gamma distribution, and utility parameters were assumed to Beta distribution (48).

**Table 1:** Key input parameters to our model and ranges of the sensitivity analyses.

**Input parameters**	**Base case value**	**Lower bound**	**Upper bound**	**Distribution**	**Source**
**Clinical input**				
PFS survival model of nivolumab	Nivolumab PFS survival data	–	–	Fixed in model	ATTRACTION-3 trial
PFS survival model of chemotherapy	Chemotherapy PFS survival data	–	–	Fixed in model	ATTRACTION-3 trial
OS survival model of nivolumab	Nivolumab OS survival data	–	–	Fixed in model	ATTRACTION-3 trial
OS survival model of chemotherapy	Chemotherapy OS survival data	–	–	Fixed in model	ATTRACTION-3 trial
**Utility input**					
PFS	0.74	0.59	0.89	Beta	(34, 37)
PD	0.58	0.46	0.70	Beta	(34, 37)
**Drug acquisition**					
Nivolumab (Bristol-Myers Squibb) per 240 mg	$3,614.08	$2,891.26	$4,336.90	Gamma	National Health Commission of China
Docetaxel (Bristol-Myers Squibb) per 20 mg	$997.19	$797.75	$1,196.63	Gamma	National Health Commission of China
paclitaxel (Aventis Pharma S A) per 40 mg	$459.60	$367.68	$551.52	Gamma	National Health Commission of China
**Drug administration**				Gamma	
Preventive medication per administered intravenously	$93.93	$75.14	$112.72	Gamma	Local medical data
Infusion fee per administered intravenously	$1.86	$1.49	$2.23	Gamma	Local medical data
Hospitalization fee per administered intravenously	$39.14	$31.31	$46.97	Gamma	Local medical data
**Laboratory tests and scans**					
ECG	$4.23	$3.38	$5.07	Gamma	Fujian Provincial Health Commission, (18, 43)
Hematology	$3.91	$3.13	$4.70	Gamma	Fujian Provincial Health Commission, (18, 43)
Serum chemistry	$28.18	$22.54	$33.81	Gamma	Fujian Provincial Health Commission, (18, 43)
Urinalysis	$4.70	$3.76	$5.64	Gamma	Fujian Provincial Health Commission, (18, 43)
Coagulation parameters	$10.42	$8.34	$12.50	Gamma	Fujian Provincial Health Commission, (18)
Thyroid function	$23.48	$18.79	$28.18	Gamma	Fujian Provincial Health Commission, (18, 43)
Pulmonary function tests	$61.05	$48.84	$73.26	Gamma	Fujian Provincial Health Commission, (18)
HBV and HCV serology	$11.28	$11.28	$19.12	Gamma	Fujian Provincial Health Commission, (18)
HBV DNA	$23.64	$23.64	$62.78	Gamma	Fujian Provincial Health Commission, (18)
Radiologic images	$435.58	$234.82	$919.69	Gamma	Fujian Provincial Health Commission, (18, 43)
**Treatment-emergent AE (grade 3–5) in nivolumab group**					
Rash	$80.00	$60.00	$100.00	Gamma	NCCN Clinical Practice Guidelines in Oncology (30)
Diarrhea	$14,000.00	$8,000.00	$20,000.00	Gamma	NCCN Clinical Practice Guidelines in Oncology (30)
Decreased appetite	$825.00	$150.00	$1,500.00	Gamma	NCCN Clinical Practice Guidelines in Oncology (30)
Stomatitis	$2,550.00	$100.00	$5,000.00	Gamma	NCCN Clinical Practice Guidelines in Oncology (30)
Nausea	$800.00	$100.00	$1,500.00	Gamma	NCCN Clinical Practice Guidelines in Oncology (30)
Arthralgia	$350.00	$100.00	$600.00	Gamma	NCCN Clinical Practice Guidelines in Oncology (30)
Neutrophil count decreased	$1,575.00	$150.00	$3,000.00	Gamma	NCCN Clinical Practice Guidelines in Oncology (30)
Anemia	$5,500.00	$1,000.00	$10,000.00	Gamma	NCCN Clinical Practice Guidelines in Oncology (30)
White blood cell count decreased	$1,575.00	$150.00	$3,000.00	Gamma	NCCN Clinical Practice Guidelines in Oncology (30)
Neutropenia	$1,575.00	$150.00	$3,000.00	Gamma	NCCN Clinical Practice Guidelines in Oncology (30)
Peripheral sensory neuropathy	$15,000.00	$10,000.00	$20,000.00	Gamma	NCCN Clinical Practice Guidelines in Oncology (30)
Febrile neutropenia	$2,650.00	$300.00	$5,000.00	Gamma	NCCN Clinical Practice Guidelines in Oncology (30)
Neuropathy peripheral	$15,000.00	$10,000.00	$20,000.00	Gamma	NCCN Clinical Practice Guidelines in Oncology (30)
**Treatment-emergent AE (grade 3–5) in chemotherapy group**					
Rash	$35.00	$20.00	$50.00	Gamma	Expert consensus of clinical practices
Diarrhea	$312.50	$25.00	$600.00	Gamma	Expert consensus of clinical practices
Decreased appetite	$825.00	$150.00	$1,500.00	Gamma	Expert consensus of clinical practices, (35)
Stomatitis	$125.00	$50.00	$200.00	Gamma	Expert consensus on the diagnosis and prevention of acute oral mucositis caused by antitumor therapy
Nausea	$350.00	$100.00	$600.00	Gamma	CSCO guidelines for the prevention and treatment of antitumor treatment-related nausea and vomiting, (35)
Arthralgia	$0.00	$0.00	$0.00	Gamma	Expert consensus of clinical practices
Neutrophil count decreased	$1,575.00	$150.00	$3,000.00	Gamma	Expert consensus on the diagnosis and treatment of neutropenia caused by tumor chemotherapy, (37)
Anemia	$275.00	$50.00	$500.00	Gamma	CSCO clinical practice guidelines for tumor-associated anemia, (34)
White blood cell count decreased	$1,575.00	$150.00	$3,000.00	Gamma	Expert consensus on the diagnosis and treatment of neutropenia caused by tumor chemotherapy, (44)
Neutropenia	$1,575.00	$150.00	$3,000.00	Gamma	Expert consensus on the diagnosis and treatment of neutropenia caused by tumor chemotherapy, (45)
Peripheral sensory neuropathy	$25.00	$0.00	$50.00	Gamma	ASCO clinical practice guidelines, (43)
Febrile neutropenia	$2,650.00	$300.00	$5,000.00	Gamma	Expert consensus on the diagnosis and treatment of neutropenia caused by tumor chemotherapy, (37)
Neuropathy peripheral	$25.00	$0.00	$50.00	Gamma	ASCO clinical practice guidelines
**Terminal cost**				
Expenditure on funeral	$4,517.85	$3,614.28	$5,421.42	Gamma	Local data
Discount rate	0.05	0	0.08	Fixed in model	(46)

*OS, overall survival; PFS, progression-free survival; PD, progressive disease; AE, adverse events; NCCN, National Comprehensive Cancer Network; ASCO, American Society of Clinical Oncology; CSCO, Chinese Society of Clinical Oncology*.

**Table 2:** Laboratory tests, scans and treatment-emergent grade3–5 AE details.

**Input parameters**	**Test/scans/treatment details**	**Source**
**Laboratory tests and scans**		
ECG	12-lead ECG	Fujian Provincial Health Commission, (18, 43)
Hematology	Red blood cell count, hemoglobin, platelet count, auto-cell count, neutrophil count, lymphocyte count	Fujian Provincial Health Commission, (18, 43)
Serum chemistry	ALT, AST, GGT, total bilirubin, direct bilirubin, AKP, blood urea nitrogen or urea (preferably blood urea nitrogen>, total protein, albumin, creatine, blood sugar, lactate dehydrogenase, K + ~ Na +, Ca2+, Mg2+, Cl-	Fujian Provincial Health Commission, (18, 43)
Urinalysis	White blood cells, red blood cells, urine protein	Fujian Provincial Health Commission, (18, 43)
Coagulation parameters	APTT, PT, FIB, TT, INR	Fujian Provincial Health Commission, (18)
Thyroid function	TSH, FT3 and FT4	Fujian Provincial Health Commission, (18, 43)
Pulmonary function tests	Spirometry and assessment of diffusion capacity	Fujian Provincial Health Commission, (18)
HBV and HCV serology	HBsAg, HBcAb, and HCV antibody	Fujian Provincial Health Commission, (18)
HBV DNA	HBV DNA	Fujian Provincial Health Commission, (18)
Radiologic images	Contrast-enhanced CT or MRI for neck, chest, and abdomen	Fujian Provincial Health Commission, (18, 43)
**Treatment-emergent AE (grade 3–5) in nivolumab group**		
Rash	Glucocorticoid therapy, supplemented with proton pump inhibitors to prevent gastrointestinal reactions	NCCN Clinical Practice Guidelines in Oncology (30)
Diarrhea	1. Perform blood routine, liver and kidney function, electrolytes, stool routine, stool culture, thyroid function, abdominal and pelvic enhanced CT, colonoscopy, etc. 2. Nutritional support 3. Glucocorticoid therapy, if glucocorticoid therapy is invalid within 48 h or worsening, consider adding infliximab while continuing to use glucocorticoids	NCCN Clinical Practice Guidelines in Oncology (30)
Decreased appetite	Megestrol, nutritional support	NCCN Clinical Practice Guidelines in Oncology (30)
Stomatitis	Mouthwash, anti-infection, nutritional support	NCCN Clinical Practice Guidelines in Oncology (30)
Nausea	Antiemetic treatment, nutritional support	NCCN Clinical Practice Guidelines in Oncology (30)
Arthralgia	Glucocorticoid therapy, if glucocorticoid therapy fails, other immunosuppressive drugs such as infliximab, methotrexate, sulfasalazine, or leflunomide may be considered	NCCN Clinical Practice Guidelines in Oncology (30)
Neutrophil count decreased	G-CSF	NCCN Clinical Practice Guidelines in Oncology (30)
Anemia	Blood transfusion, glucocorticoid therapy, if glucocorticoid therapy fails, immunosuppressant can be given	NCCN Clinical Practice Guidelines in Oncology (30)
White blood cell count decreased	G-CSF	NCCN Clinical Practice Guidelines in Oncology (30)
Neutropenia	G-CSF	NCCN Clinical Practice Guidelines in Oncology (30)
Peripheral sensory neuropathy	Close monitoring of neurological symptoms and respiratory function; immunoglobulin or plasma exchange; glucocorticoid therapy	NCCN Clinical Practice Guidelines in Oncology (30)
Febrile neutropenia	G-CSF; antibiotics	NCCN Clinical Practice Guidelines in Oncology (30)
Neuropathy peripheral	Close monitoring of neurological symptoms and respiratory function; immunoglobulin or plasma exchange; glucocorticoid therapy	NCCN Clinical Practice Guidelines in Oncology (30)
**Treatment-emergent AE (grade 3–5) in chemotherapy group**		
Rash	Dexamethasone, antihistamines	Expert consensus of clinical practices
Diarrhea	Anti-diarrheal treatment	Expert consensus of clinical practices
Decreased appetite	Megestrol, nutritional support	Expert consensus of clinical practices, (35)
Stomatitis	Mouthwash, anti-infective treatment if necessary	Expert consensus on the diagnosis and prevention of acute oral mucositis caused by antitumor therapy
Nausea	Antiemetic treatment	CSCO guidelines for the prevention and treatment of antitumor treatment-related nausea and vomiting, (35)
Arthralgia	/	Expert consensus of clinical practices
Neutrophil count decreased	G-CSF	Expert consensus on the diagnosis and treatment of neutropenia caused by tumor chemotherapy, (37)
Anemia	Iron supplementation, blood transfusion therapy	CSCO clinical practice guidelines for tumor-associated anemia, (34)
White blood cell count decreased	G-CSF	Expert consensus on the diagnosis and treatment of neutropenia caused by tumor chemotherapy, (44)
Neutropenia	G-CSF	Expert consensus on the diagnosis and treatment of neutropenia caused by tumor chemotherapy, (45)
Peripheral sensory neuropathy	Nutritional nerve therapy	ASCO clinical practice guidelines, (43)
Febrile neutropenia	G-CSF; antibiotics	Expert consensus on the diagnosis and treatment of neutropenia caused by tumor chemotherapy, (37)
Neuropathy peripheral	Nutritional nerve therapy	ASCO clinical practice guidelines

*ECG, electrocardiogram; HBV, hepatitis B virus; HCV, hepatitis C virus; DNA, deoxyribonucleic acid; ALT, alanine aminotransferase; AST, aspartate aminotransferase; GGT, gamma glutamyl transpeptidase; AKP, alkaline phosphatase; APTT, activated partial thromboplastin time; PT, prothrombin time; FIB, fibrinogen; TT, thrombin time; INR, international standard ratio; TSH, thyroid stimulating hormone; FT3, free triiodothyronine; FT4, free thyroxine; CT, computed tomography; MRI, magnetic resonance imaging.; AE, adverse events; G-GSF, granu1ocyte colony-stimu1ating factor; NCCN, National Comprehensive Cancer Network; ASCO, American Society of Clinical Oncology; CSCO, Chinese Society of Clinical Oncology*.

### Statistical Analysis

In our model, the cost and health outcomes of the three mutually exclusive health states, as well as deterministic and probabilistic sensitivity analyses, were computed using Excel 2016. The clinical efficacy and safety data of second-line therapy for advanced ESCC were obtained from the ATTRACTION-3 trial. In the ATTRACTION-3 trial, statistical analyses were completed using SAS 9.4. OS and PFS were estimated using the Kaplan-Meier method, with a two-sided, 0.05 significance level, log-rank test. We performed a survival analysis similar to the ATTRACTION-3 trial for estimating the survival curve. Statistical analyses were undertaken using SPSS 26.0. OS was estimated using the Kaplan-Meier method, with a two-sided, 0.05 significance level, log-rank test. Further, PFS was estimated using the life table method.

## Results

### Base-Care Analysis

The median OS in the ATTRACTION-3 study was 10.9 months for the nivolumab group and 8.4 months for the chemotherapy group (stratified log-rank, *P* = 0.019) up to the data cut-off point (November 12, 2018). The median PFS in the nivolumab group was 1.7 months, compared to 3.4 months in the paclitaxel or docetaxel treatment group. The ATTRACTION-3 trial also reported details of the survival rate. The 12-month OS in the nivolumab group was 47%, compared to 34% in the chemotherapy group. The 18-month OS in the nivolumab group was 31%, while that in the chemotherapy group was 21%. The 6-month PFS in the nivolumab group was 24%, compared to 17% in the chemotherapy group. The 12-month PFS in the nivolumab group was 12%, while that in the chemotherapy group was 7%.

**Figure 2:**
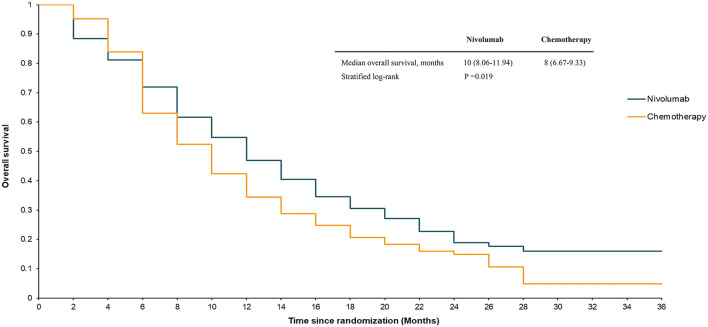
Estimated overall survival curve for the ATTRACTION-3 trial.

**Figure 3:**
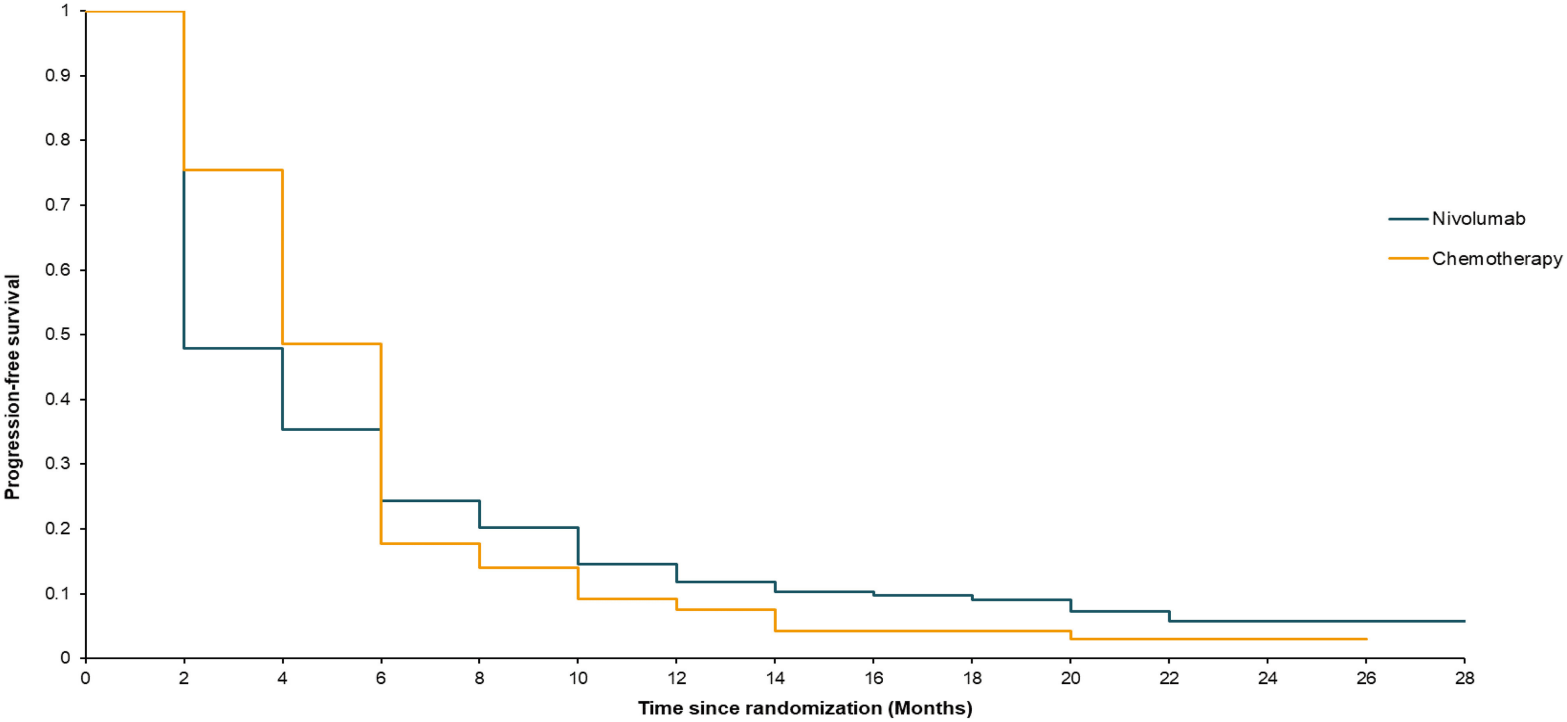
Estimated progression survival curve for the ATTRACTION-3 trial.

Our model simulated a hypothetical sample of 419 patients. The model's survival analysis results were remarkably close to the actual clinical trial data. The median OS in the model was 10 months for nivolumab and 8 months for chemotherapy (stratified log-rank, *P* = 0.019) (Figure 2). The nivolumab group had a PFS rate of 1.92 months, compared to 3.89 months in the chemotherapy group (Figure 3). Furthermore, the survival rate statistics were remarkably similar to the actual clinical study data. The 12-month OS in the nivolumab group was 46.9%, compared to 34.4% in the chemotherapy group. The 18-month OS rate was 30.5% in the nivolumab group and 20.7% in the chemotherapy group. The 6-month PFS was 24.3% in the nivolumab group, while it was 17.7% in the chemotherapy group. The 12-month PFS was 11.9% in the nivolumab group, while it was 7.6% in the chemotherapy group.

During the 3-year study period, nivolumab immunotherapy's cost was US$ 57,624.92 and exceeded paclitaxel/docetaxel chemotherapy's cost of US$ 20,668.11, by US$ 36,956.81. Interestingly, out of this incremental cost, that of drug acquisition was US$ 39,467.00, which exceeded the total incremental cost (US$ 36,956.81). Besides, in the PD stage, the nivolumab group's cost per patient was US$ 45, higher than that of the chemotherapy group, despite the fact that the costs of nivolumab treatment were lower than those of paclitaxel/docetaxel chemotherapy in terms of drug administration, laboratory tests, radiologic images, terminal, and treatment-related AEs. Nivolumab immunotherapy resulted in an improvement of 0.28 QALY (0.80 vs. 0.52) per patient compared with paclitaxel/docetaxel chemotherapy. The ICER for the nivolumab group vs. the chemotherapy group was estimated to be US$ 132,029.46/QALY (Table 3).

**Table 3:** Results of our model.

**Results**	**Nivolumab group**	**Chemotherapy group**
Total costs	$57,624.92	$20,668.11
QALYs	0.80	0.52
ICER, $/QALYs	$132,029.46	–

*QALYs, quality-adjusted life-years; ICER, incremental cost-effectiveness ratio*.

### Sensitivity Analyses

#### Deterministic Sensitivity Analyses

**Figure 4:**
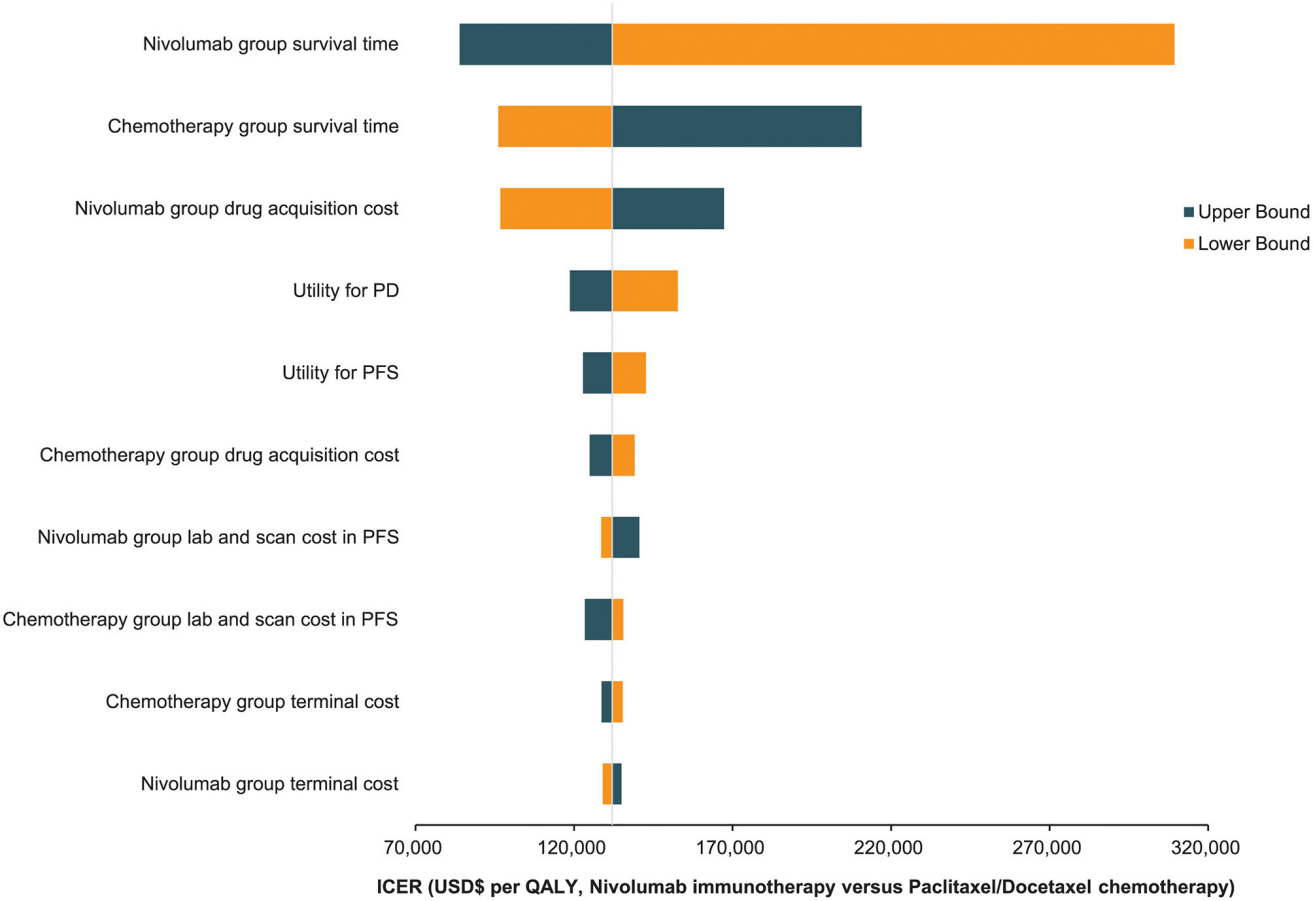
The Top 10 most influencing parameters in Tornado diagram.

The findings of the one-way deterministic sensitivity analyses revealed that the model was most sensitive to the nivolumab group's survival time. The model was heavily influenced by the following parameters: chemotherapy group survival time, nivolumab group medication acquisition cost, and utility scores. The top 10 most influencing parameters are presented in a tornado diagram (Figure 4). The ICER of nivolumab did not decrease below US$ 80,000/QALY despite the varied ranges for each variable. Nivolumab's drug acquisition must be cut by 53.50% to obtain a more favorable cost-effectiveness under the threshold cost of US$ 37,623.39/QALY, which the Chinese populace can afford.

#### Probabilistic Sensitivity Analyses

**Figure 5:**
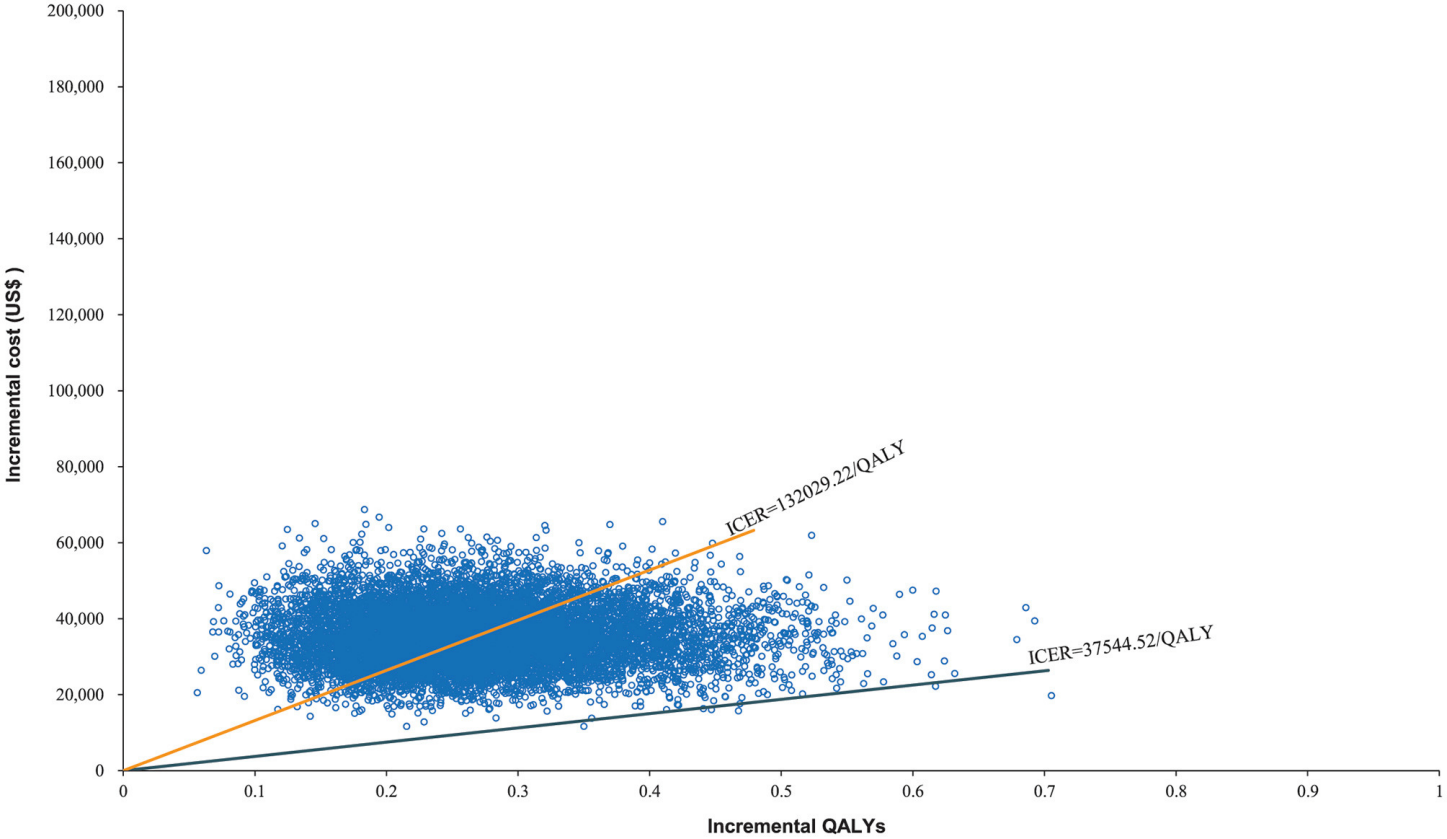
Scatter plot of Monte Carlo sensitivity analysis.

**Figure 6:**
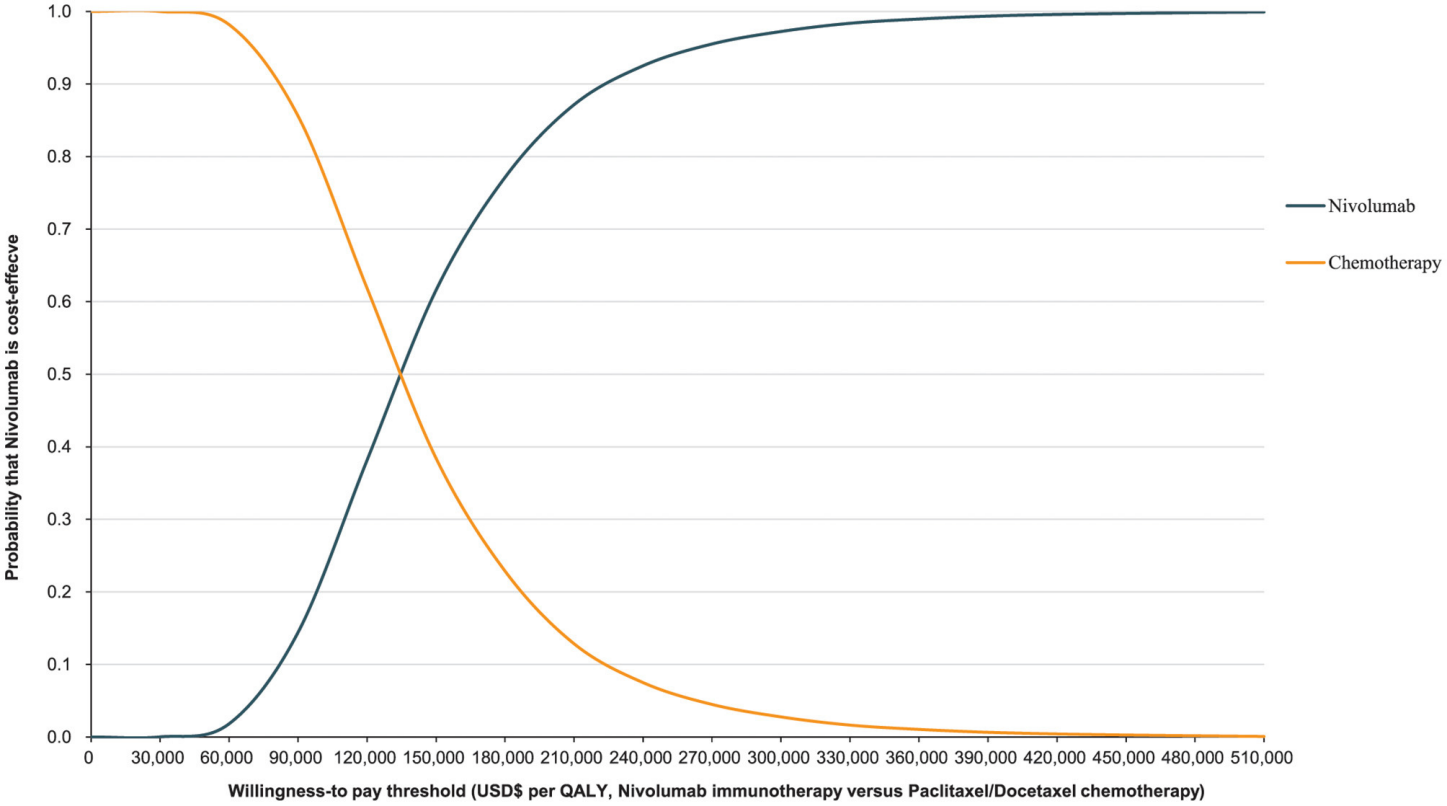
Cost-effectiveness acceptability curve for nivolumab immunotherapy vs. paclitaxel or docetaxel chemotherapy.

The World Health Organization places the willingness-to-pay (WTP) threshold at three times the GDP per capita (49). In 2021, the GDP per capita of the Chinese population was US$ 12,551, making the WTP threshold US$ 37,653/QALY. The Monte Carlo probabilistic sensitivity analyses revealed that the probability of nivolumab immunotherapy not being a cost-effective option when compared with paclitaxel/docetaxel chemotherapy at a WTP threshold of US$ 37,653/QALY. When the WTP threshold changed to US$ 132,029.22/QALY, the closest number to 132,029.46/QALY in simulated iterations, the probability of nivolumab immunotherapy being cost-effective when compared with paclitaxel/docetaxel chemotherapy increased to 48.02% (Figures 5, 6).

## Discussion

The costs associated with healthcare have become one of the world's most serious issues. Many scholars have developed healthcare economic evaluation models to assess the economic effects of immunotherapeutic inhibitors in antineoplastic therapy. These models all agree that in order for a therapy to be cost-effective, it must have two crucial characteristics: a lower cost and a higher effectiveness (50). This expectation was represented as extra cost and incremental QALYs in this study. Nivolumab immunotherapeutic inhibitors had a greater survival rate in advanced ESCC treatment than paclitaxel/docetaxel chemotherapy, however, they would also increase healthcare costs dramatically. Nivolumab costs US$ 132,029.46 for every extra QALY achieved when compared to chemotherapy. From the Chinese healthcare system perspective, this may not be a cost-effective treatment option. At the WTP threshold of US$ 132,029.22/QALY, the probabilistic sensitivity analysis revealed that nivolumab was not an economical alternative, with only a 48.02% chance of becoming cost-effective. Moreover, when the WTP threshold changed to US$ 37,544.52/QALY, the probability declined to 0.07%. The ICER of US$ 37,544.52/QALY is nearly the World Health Organization's recommended threshold in 2021. These findings indicate that nivolumab is in effect not a value second-line therapeutic modality in China for advanced ESCC.

Advanced ESCC is a fast and fatal disease. Even with immunotherapy, patients' quality of life suffers due to their dismal prognosis (41). The patients' lives end and their families descend into poverty due to the cost of treating the illness. What makes nivolumab less cost-effective than chemotherapy? Surprisingly, we found that the incremental cost of nivolumab (US$ 39,467.00) was higher than the total incremental cost of its use (US$ 36,956.81). This means that nivolumab acquisition is much costlier than chemotherapy; reducing this immune inhibitor's price can significantly improve the cost-effectiveness of its use. This finding was supported by the one-way sensitivity analyses. After nivolumab group's survival time and chemotherapy group's survival time, the drug acquisition cost of the nivolumab group was the third parameter that had the greatest impact on our model. Although the price of nivolumab in China is cheaper than in some other countries, it must decline by 53.50% to meet the WTP threshold, which is approximately three times the Chinese population's GDP per capita.

Can nivolumab become cost-effective by improving patients' survival time? Whether nivolumab would achieve cost-effectiveness by extending patients' survival time sufficiently so that the cost gap between nivolumab and chemotherapy would be recovered during long-term treatment is unknown. In such cases, PD-1 immunotherapy can provide both clinical and financial benefits in the form of prolonged survival and improved quality of life. An additional two clinical trials [KEYNOTE-181 (51) and ESCORT (52)] also demonstrated that PD-1 inhibitors would improve clinical efficacy in comparison to chemotherapy in advanced ESCC treatment. However, if medical cost is constant, such improvement is not enough to make PD-1 inhibitors more cost-effective than chemotherapy. One-way sensitivity evaluations in our model revealed that if nivolumab becomes a more cost-effective therapy alternative than chemotherapy, the survival time of the nivolumab group would have to be prolonged two additional times. In that case, ICER would achieve US$ 34,148.47/QALY, which is less than three times the Chinese population's GDP per capita. Although similar to the ATTRACTION-3 trial, these two studies found that patients who received PD-1 inhibitors had prolonged survival time than those who received chemotherapy. However, the improvement in survival time as a result of PD-1 inhibitors for advanced ESCC immunotherapy was insufficient. The KEYNOTE-181 trial on 628 patients, comparing pembrolizumab with paclitaxel/docetaxel/irinotecan chemotherapy, showed that the median OS of pembrolizumab (9.3 months) was longer than that of chemotherapy (6.7 months); however, the median PFS at 2.6 months was shorter than 3.0 months in the case of chemotherapy. The ESCORT trial of camrelizumab and docetaxel/irinotecan chemotherapy on 448 patients reported that the median OS with camrelizumab (8.3 months) was longer than that with chemotherapy (6.2 months). The median PFS, in either case, was 1.9 months.

Additionally, nivolumab, pembrolizumab, camrelizumab, and most PD-1 inhibitors (spartalizumab, toripalimab, sintilimab, etc.) are fully human IgG4 monoclonal antibodies. This means their IgG isotypes or mutants with nullified effector functions are similar (53). It seems that the PD-1 inhibitors may still have similar clinical efficacy in advanced ESCC treatment until pharmaceutical production technology does not change. Fortunately, new pharmaceutical manufacturing technologies are being developed to produce a series of PD-1 inhibitors (PD-1/CTLA-4, PD-1/CD47, PD-1/LAG-3, etc.), which could potentially be used in the future to treat advanced ESCC. Meanwhile, in 2019, there was a remarkable medical market revolution in China. The General Office of the State Council of the People's Republic of China implemented a price negotiation of the National Reimbursement Drug List to deal with the challenges of ever-increasing medical expenditures, make drugs more affordable for patients, and make steady efforts to reform the drug procurement system. In 2021, the price of camrelizumab declined sharply from US$ 3,100/200 milligrams to US$ 458/200 milligrams. Driven by the “price reduction and volume increment,” if a growing number of PD-1 inhibitors with lower prices than nivolumab become available, the price of nivolumab may be reduced in the future due to market competition. In our model, lowering the price of nivolumab by 53.50% might make it a cost-effective and affordable therapy choice for advanced ESCC patients in the Chinese population.

The ESCORT trial and KEYNOTE-181 trial also reported an economic evaluation of the cost-effectiveness of PD-1 inhibitors by developing a Markov model (35, 43). The findings suggested that in 2019, camrelizumab immunotherapy may not have been a more cost-effective therapeutic choice for advanced ESCC than chemotherapy. Camrelizumab incurred an incremental cost of US$ 24,539 and an effect of 0.283 QALYs compared with docetaxel/irinotecan chemotherapy, whereas the ICER incurred US$ 86,745/QALY. Further, in 2021, pembrolizumab immunotherapy may not have been a more cost-effective therapeutic option for advanced ESCC than chemotherapy. Pembrolizumab demonstrated an incremental cost of US$ 19,054.61 and an effect of 0.09 QALYs compared with paclitaxel/docetaxel/irinotecan chemotherapy, whereas the ICER incurred US$ 202,708.62/QALY. Although the ESCORT trial and KEYNOTE-181 trial have many similarities to the ATTRACTION-3 trial and the cost-effectiveness analysis results are consistent with our findings, the modeling methods are quite different. Initially, we attempted to establish a Markov model for cost-effectiveness analysis. By digitizing the OS and PFS curves from the ATTRACTION-3 trial, we were able to determine time and survival probability using the GetData Graph Digitizer. According to the lowest Akaike information criteria and Bayesian information criterion values, we found that a 2-parameter Weibull distribution was the best-fitting distribution model for the pseudo-individual patient data. However, we found a high degree of bias in the results were obtained using the Markov model compared with the actual ATTRACTION-3 trial results. For example, PFS's transition probability to death was not rigorous; the Markov model needs to calculate the transition probability between different health stages, but PFS's transition probability to death could not be calculated. Therefore, we had to utilize the general Chinese population's mortality rate as the transition probability of PFS to death, an approach also employed in other studies (35, 54). The median survival of patients with advanced ESCC is only 8–10 months, and the expected 5-year survival rate is less than 5% (5). Patients with advanced esophageal cancer had a greater mortality rate than the general population, even at the PFS stage (55, 56). Although the general population's mortality rate is a fixed value, the death rate varies in each model cycle because of the decreased functioning and worsening symptoms (41). Meanwhile, the 2-parameter Weibull distribution showed substantial divergence from the original survival curves. This divergence was evident for both the OS and PFS curves. In this study, patients had a significantly different survival rate in the 2-parameter Weibull distribution than that observed in the ATTRACTION-3 trial, as the trial's time horizon was defined as 3 years. The same divergence was also observed in the Markov model evaluation of the ESCORT trial (35). We carefully checked the references and concluded that this distribution could provide an appropriate fit for the longer-term extrapolation of clinical trial data, but may have inherent uncertainty in the short-term assessment of the survival curve (57, 58). Some previously reported models for the treatment of Non-small cell lung cancer, hepatocellular carcinoma, and melanoma included curve extrapolation (47, 59, 60). When the model simulates time beyond the follow-up period, the distribution of the number of people in each health state cannot be obtained directly from the survival curve. Therefore, a parametric method was used to calculate the survival function. This method assumes that the survival time obeys a particular parametric distribution. However, patients with advanced ESCC have a short-term disease progression and mortality rate, and clinical trials can simulate the disease transition in mutually exclusive health stages without extrapolating the survival data. Therefore, we rebuilt the cost-effectiveness model using the partitioned survival model and accurate data, but did not perform extrapolation beyond the ATTRACTION-3 trial's follow-up period. We believe that this improvement may be more suitable for simulating the treatment of advanced ESCC.

To our knowledge, few studies have empirically investigated the cost-effectiveness of immunotherapy inhibitors for advanced ESCC. Some of the previous studies analyzed medical and economic data sourced from other studies to reach their conclusions. Therefore, the study's main strength is that it directly compared nivolumab immunotherapy to paclitaxel/docetaxel chemotherapy utilizing original, published trial data as well as clinical expenses, financial data, and utility values gathered in the course of clinical practice. As the price of PD-1 inhibitors decreased significantly after the implementation of the price negotiation of the National Reimbursement Drug List, it became necessary to evaluate the scope of price reduction for both pharmaceutical enterprises and the government. Our model's survival analysis results are extremely similar to the actual data from the ATTRACTION-3 trial. Therefore, the economic evaluation results of our model are reliable and may have reference value for subsequent policy practice.

There are a few limitations in this study. First, our model essentially relied on the ATTRACTION-3 trial; however, patients participating in clinical trials are different from those in real-world clinical practices. This difference might introduce biases in the cost-effectiveness evaluation (61). Due to the lack of global/domestic multicenter phase 4 or real-world studies, phase 3 trials may provide the best clinical evidence available thus far for cost-effectiveness analysis in the treatment of advanced ESCC. Although the frequency of the tests in clinical trials differs from real world experience, which would increase the cost in our model, the model provided a reasonable, albeit imperfect, approximation to the real-world clinical benefit observed in the clinical trials. Second, the model based on the survival analysis did not make the assumption that survival time follows a specific parametric distribution. Although we believe that survival analysis for survival curves estimation has a good fit for the survival curves in the ATTRACTION-3 trial, this method also may increase the complexity of the model. Therefore, in the long-term extrapolation of survival time, the modeling findings may not accurately represent the disease course. The survival curve extrapolation in our model may be improved by incorporating another phase 4 trial or real-world study in our model. Third, AE-related expenditures for grades 1 to 2 were not included in the model, which may have undermined the economic evaluation results. However, as suggested by the deterministic sensitivity analyses, AE-related costs are a minor component of the total cost. Perhaps, collecting more survival follow-up information and safety data in future studies to fully reproduce the clinical course of nivolumab immunotherapy vs. chemotherapy in advanced ESCC may result in a more accurate economic evaluation.

## Conclusion

This study analyzed the cost-effectiveness of nivolumab immunotherapy compared with docetaxel or paclitaxel in the treatment of advanced ESCC. Nivolumab is clinically beneficial, but such a benefit cannot offset the expensive medical cost, which leads to the conclusion that nivolumab is not a cost-effective therapy option in the treatment of advanced ESCC when compared to chemotherapy. A substantial reduction in nivolumab's drug acquisition cost would be necessary to make its use cost-effective for this immunotherapy. A substantial reduction in nivolumab's price may be achieved through changes in the PD-1 inhibitor market competition in China and the price negotiation of the National Reimbursement Drug List.

## Data Availability Statement

The original contributions presented in the study are included in the article/supplementary material, further inquiries can be directed to the corresponding author/s.

## Author Contributions

Y-tL: methodology, original draft writing, software, and data curation. T-xL: writing, reviewing, and editing. JC: software, investigation, and data curation. CW: software, visualization, and data curation. YC: conceptualization, editing, and supervision. All authors contributed to the article and approved the submitted version.

## Funding

This study was supported by the Startup Fund for Scientific Research, Fujian Medical University (Grant number: 2019QH1201).

## Conflict of Interest

The authors declare that the research was conducted in the absence of any commercial or financial relationships that could be construed as a potential conflict of interest.

## Publisher's Note

All claims expressed in this article are solely those of the authors and do not necessarily represent those of their affiliated organizations, or those of the publisher, the editors and the reviewers. Any product that may be evaluated in this article, or claim that may be made by its manufacturer, is not guaranteed or endorsed by the publisher.

## Abbreviations

ESCC: esophageal squamous cell carcinoma
PD-1 inhibitor: anti-programmed death 1
PD stage: progressive disease
QALYs: quality-adjusted life years
ICER: incremental cost-effectiveness ratio
AE: adverse events
PFS: progression-free survival
OS: overall survival
WTP: willingness-to-pay.
